# Factors affecting RIG-I-Like receptors activation - New research direction for viral hemorrhagic fevers

**DOI:** 10.3389/fimmu.2022.1010635

**Published:** 2022-09-29

**Authors:** Paulina Małkowska, Paulina Niedźwiedzka-Rystwej

**Affiliations:** ^1^ Doctoral School, University of Szczecin, Szczecin, Poland; ^2^ Institute of Biology, University of Szczecin, Szczecin, Poland

**Keywords:** RLR, immune activation, signaling, signaling pathways, VHF, *Lagovirus europaeus*, interferon, MAVS

## Abstract

Viral hemorrhagic fever (VHF) is a term referring to a group of life-threatening infections caused by several virus families (*Arenaviridae*, *Bunyaviridae*, *Filoviridae* and *Flaviviridae*). Depending on the virus, the infection can be mild and can be also characterized by an acute course with fever accompanied by hypervolemia and coagulopathy, resulting in bleeding and shock. It has been suggested that the course of the disease is strongly influenced by the activation of signaling pathways leading to RIG-I-like receptor-dependent interferon production. RIG-I-like receptors (RLRs) are one of two major receptor families that detect viral nucleic acid. RLR receptor activation is influenced by a number of factors that may have a key role in the differences that occur during the antiviral immune response in VHF. In the present study, we collected data on RLR receptors in viral hemorrhagic fevers and described factors that may influence the activation of the antiviral response. RLR receptors seem to be a good target for VHF research, which may contribute to better therapeutic and diagnostic strategies. However, due to the difficulty of conducting such studies in humans, we suggest using *Lagovirus europaeus* as an animal model for VHF.

## 1 Introduction

There are two levels of immune system functions: innate and adaptive, although in practice there is much interaction between them. Innate immunity is a rapid, but non-specific early warning system for global immunity (1). If pathogens circumvented physical barriers such as the skin or mucus membranes, biochemical mechanisms quickly identify any “non-self” molecules (2). The host recognizes conserved molecular structures known as pathogen-associated molecular patterns (PAMPs) that are present in pathogens. These PAMPs are sensed by the host’s pattern recognition receptors (PRRs) which are expressed on innate immune cells (3, 4). Elimination of pathogens is caused *via* the activation of complex signaling pathways which induce inflammatory responses mediated by various cytokines and chemokines (4).

PRRs are responsible for the initiation of immune responses in mammals. They are located in subcellular compartments (cellular and endosomal membranes), the cytosol and extracellularly, in secreted forms present in the bloodstream and interstitial fluids (5). PRRs recognize PAMPs, such as lipopolysaccharide in gram-negative bacteria or intercellular single- and double-stranded viral RNA (6, 7). Different kinds of PRRs are specialized to recognize different PAMPs. To date, five classes of PRRs, such as Toll-like receptors (TLRs), RIG-I-like receptors (RLRs), NOD-like receptors (NLRs), C-type lectin-like receptors (CRLs) and AIM-2-like receptors (ARLs), have been characterized (8–12). The recognition of viral infection involves the cytosolic DNA sensor cyclic GMP–AMP synthase (cGAS), TLRs and RLRs (9, 13). Of the TLRs that we know, TLR3 recognizes viral dsRNA, TLR7 and human TLR8 identify viral ssRNA and TLR9 detects viral DNA. These TLRs are located in endosomal compartments. These receptors activate the signaling pathways that lead to the production of type I interferons and inflammatory cytokines (14).

## 2 RIG-I-like receptors

RIG-I-like receptors are a family of helicases, that function as cytoplasmic sensors of pathogen-associated molecular patterns (PAMPs) within viral RNA. They recognized intracellular single- and double-stranded RNA that is introduced to the cytosol during viral infection and replication. To date, three RLR members have been identified: RIG-I (retinoic acid-inducible gene I), MDA5 (melanoma differentiation associated factor 5), and LGP2 (laboratory of genetics and physiology 2 and a homolog of mouse D11lgp2) (15). The RLRs signal production of type 1 interferon (IFN) and antiviral gene expression that cause an intracellular immune response to control virus infection (15).

RIG-I is encoded by the *DDX58* gene identified in 1997 by Sun (16). Yoneyama et al. (7) confirmed the involvement of this gene in the induction of antiviral response by searching the cDNA library for a factor that activates IFN-β expression. The gene encoding MDA5, also called Helicard, *IFIH1* (interferon induced with helicase C domain 1) or *RH116* (RNA helicase 116), was discovered in 2002 by subtractive hybridization (17). It is involved in the induction of interferon expression during viral infection (18). The *DHX58* gene encoding LGP2 protein was identified as a factor that is expressed in breast cancer cells (19). The LGP2 receptor modifies viral RNA by releasing proteins from ribonucleoprotein (RNP) complexes and altering the structure of RNA, which enables a recognition of viral dsRNA by RIG-I and MDA5 receptors (20).

**Figure 1:**
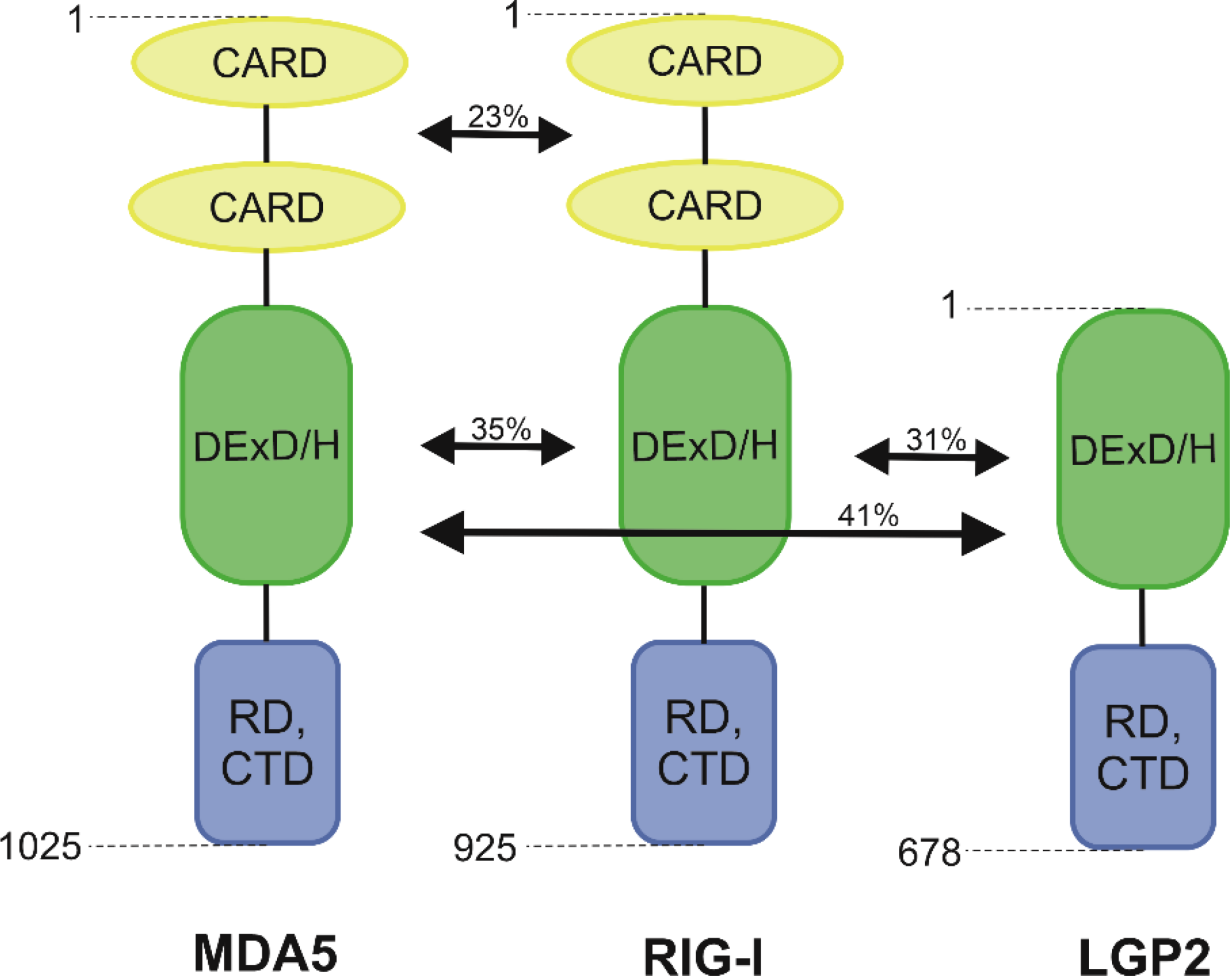
Schematic structure of RIG-I-like receptors.

RIG-I and MDA5 are mostly known for their functions inside innate immune cells, such as macrophages, neutrophils, and dendritic cells, as well as in other cells like mucosal epithelial cells, but they are expressed in all types of cells (21). They are classified as ATP-dependent DExD/H box RNA helicases (22). N-terminal portion of RIG-I encompassed the signalling domain with 2 repeats of the Caspase activation and recruitment domain (CARD). The largest portion of RIG-I is the helicase domain DExD/H. The C-terminal domain (CTD) consists of four conserved cysteines (C810, C813, C864, C869) linked by zinc or, less commonly, a mercury atom. These are the major structural and functional components of this protein that is responsible for transmitting the signal that activates the antiviral response by binding dsRNA and ssRNA 5’-triphosphate (23, 24). The activation of RIG-I receptor expression is only possible when the appropriate ligand interacts with the CTD domain (9). RIG-I also contains a repressor domain (RD) that is mapped to a region partially overlapping the RNA-binding domain (25). Receptors MDA5 consists of all these domains, but its RNA recognition domain has a significantly low affinity for dsRNA as well as little repression (25, 26). Yoneyama et al. (7) observed that RIG-I shows 23% homology in amino acid composition for the N-terminal tandem CARD and 35% for the helicase domain (**Figure 1**). Unlike RIG-I and MDA5, LGP2 lacks the N-terminal tandem CARD, which is responsible for transmitting intracellular signals. It is supposed that the lack of CARD domains in the LGP2 prevents the recognition of viral RNA through this receptor (27).

### 2.1 Virus recognition mechanism

The recognition of viral nucleic acid in the cytoplasm is based on the detection of unmodified ssRNA and dsRNA molecules that are not naturally synthesized in host cells (9, 16) (**Table 1**). RIG-I binds short dsRNA fragments 21-27 nucleotides long resulting from digestion by RNase III, whereas MDA5 recognizes long fragments of dsRNA (>2 kbp) (9). In addition, both receptors recognize the synthetic dsRNA analog poly(I:C). Poly(I:C) of approximately 300 bp length is a ligand for the RIG-I receptor, whereas MDA5 recognizes poly(I:C) molecules of 4-8 kbp length (9, 28). Studies also showed that short 1,2-1,4 kbp viral dsRNA chains induced RIG-I receptor expression, whereas dsRNA chains of 3,4 kbp length activated MDA5 expression (25). In addition, the RIG-I receptor also distinguishes ssRNA containing a 5’ triphosphate at the 5’ end (5’ppp) (29). Pathogen recognition by RIG-I-like receptors is a complex and multistep process. Previous results indicated that the length of the viral nucleic acid chain is a major determinant of identification and ligand binding by RIG-I or MDA5. The mechanism by which these receptors discriminate between PAMP ligands in terms of their length is unclear.

**Table 1:** Ligands and viruses that associate with each RLR.

RIG-I-like receptor	PAMPs	Synthetic ligands	Example of viruses
RIG-I	• short dsRNA (<2kbp) •5’ppp dsRNA •5’ppp ssRNA	• poly(I:C) (~300bp)	Reovirus, dengue, West Nile, rotavirus, Sendai, Vesicular stomatitis, influenza A, influenza B, hepatitis C, Japanese encephalitis, ebola, Newcastle disease, Lassa virus, Epstein-Barr virus
MDA5	•long dsRNA (>2kbp)	•poly(I:C) (4-8bp)	Reovirus, dengue, West Nile, rotavirus, Sendai, mengo, encephalomyocarditis, polio, murine norovirus, Theiler
LGP2	•dsRNA •5’ppp ssRNA	–	Encephalomyocarditis, vaccinia, mengo*

*only viruses which cause that LGP2 take a role in the antiviral immune response.

**Figure 2:**
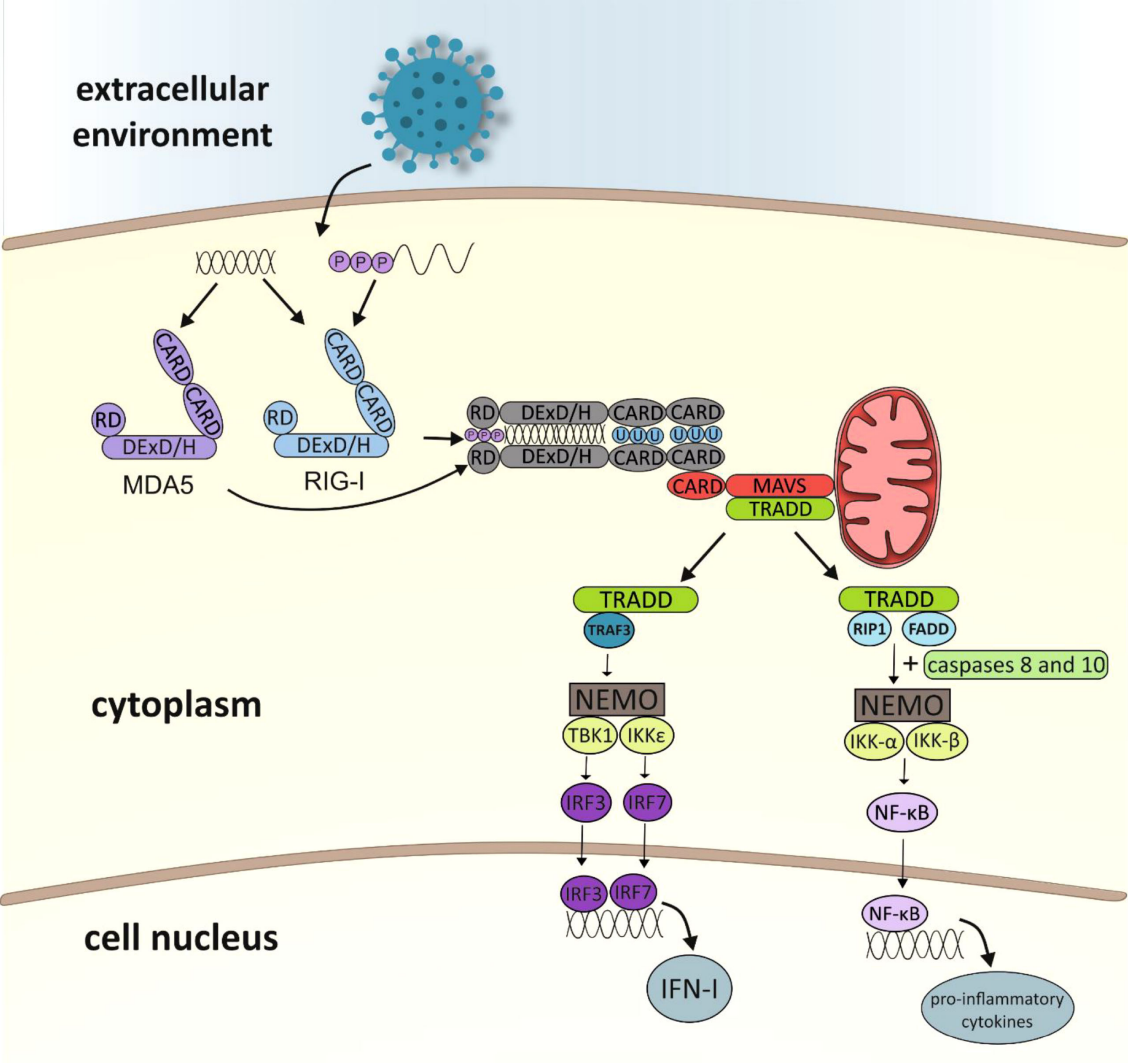
Diagram showing activation of the immune response leading to RLR receptor-dependent production of type I interferon and pro-inflammatory cytokines (CARD, caspase activation and recruitment domain; RD, repressor/regulatory domain; MDA5, melanoma differentiation associated gene 5; RIG-I, retinoic acid-inducible gene-I; MAVS, mitochondrial antiviral signaling protein; TRADD, TNFR1-associated death domain; TRAF3, TNF receptor-associated factor 3; RIP1, receptor interacting protein 1; FADD, Fas-associated death domain; NEMO, NF-κB essential modulator; NF-κB essential modulator; TBK1, TANK-binding kinase 1; IKK, IκB kinase; IRF, IFN-gene regulatory factor; NF-κB –nuclear factor κB; IFN, interferon).

Kowalinski and co-workers (30) delineated the structural biology of RIG-I receptor activation. In uninfected cells, RIG-I is thought to be in an autorepressed conformation, with the CARDs unavailable for signal transduction. The CTD mediates RNA binding to RIG-I by binding blunt-end 5′ppp-dsRNA. After connection with RNA ligand, the helicase domain of RIG-I tightly wraps around the RNA in a C-clamp-like fashion (13). Viral RNA binding to RIG-I is thought to induce conformational changes that expose the CARDs, allowing E3-ligase tripartite motif-containing 25 (TRIM25)-dependent K63 polyubiquitination of Lys172 or endogenous K63-linked polyubiquitin chain binding (31, 32). Then polyubiquitinated CARDs from RLRs interact with the CARD domain found in mitochondrial antiviral-signaling protein (MAVS which is also known as IFN-β promotor stimulator 1 [IPS-1], CARD adapter inducing IFN-β [Cardif] or virus-induced signaling adapter [VISA]). MAVS is anchored with its transmembrane domain into mitochondria. MAVS activates the adaptor protein TRADD (tumour necrosis factor receptor type 1-associated death domain protein) and triggers two alternative pathways for intracellular signal transduction: the TNF receptor-associated factor 3 (TRAF3) protein-dependent or the Fas-associated death domain (FADD) protein-dependent (33–35) (**Figure 2**). The first is based on transmitting a signal to TANK-binding kinase 1 (TBK1) and IκB kinase-ϵ (IKKϵ) (13). Then, the transcription factors IRF3 (interferon regulatory factor 3) and IRF7 (interferon regulatory factor 7) are activated and coordinate the expression of type I IFN genes (36). The second pathway involves the protein FADD, which forms a complex with RIP1 (receptor interacting protein 1) kinase *via* its death domain (DD). In addition, it also interacts with caspases 8 and 10, which activate the IKK complex (IKK-α, IKK-β and NEMO/IKKγ protein). Activated IκB undergoes phosphorylation and degradation releasing NF-κB (nuclear factor-κB). The NF-кB factor travels to the cell nucleus, where it induces the expression of pro-inflammatory cytokines (37, 38).

## 3 Mechanisms affecting RLR activity

Many factors influence the activity of RLR receptors. Mechanisms taking place inside the cell can affect both the precise operation of the signaling pathway and contribute to protein inactivation. Below, we would like to present the processes that can affect the regulation of RIG-I-like receptors (**Table 2**).

**Table 2:** Factors affecting activation of the RIG-I-like receptor-dependent signaling pathway.

Effect	Factor	Target	Mechanism	References
**Positive**	MFN1	MAVS	stimulation mitochondrial fusion and elongation	(39, 40)
	STING	MAVS	TBK1 and IRF-3 recruitment to MAVS	(39)
	Ankrd17	RIG-I, MDA5	strong interaction with MAVS by enhancing its interaction with RIG-I and MDA5	(41)
	RNF135	RIG-I	RIG-I ubiquitination	(42)
	TRIM25	RIG-I	RIG-I ubiquitination	(31)
	RAVER1	MDA5	promotion of MDA5 binding to poly(I:C)	(43)
**Negative**	USP3, USP15, USP21, CYLD	RIG-I	K63 deubiquitination	(44–47)
	RNF125	RIG-I, MDA5, MAVS	leading to degradation of RIG-I, MDA5 and MAVS	(48, 49)
	HOIL-1L	RIG-I	competing with TRIM25 for RIG-I CARD binding	(48)
	HOIP	RIG-I	promotion M1- and K48-linked polyubiquitination of TRIM25 and inducing its proteasomal degradation	(48)
	NLRX1	MAVS	competing with activated RIG-I and MDA5 for binding of the CARD domain of the MAVS	(48, 50)
	A20	TBK1, IKKi	kinase deubiquitination	(51)
	TAX1BP1	RIG-I	acting as an adaptor molecule for A20	(52)
	Atg5-Atg12	MAVS, RIG-I	interaction directly with MAVS and RIG-I through CARD domains	(53)

### 3.1 Positive regulators of RLR

#### 3.1.1 MFN1

Mitofusin 1 (MFN1) plays an important role in MAVS redistribution and is a key regulator of mitochondrial fusion forming MAVS-MFN1 complexes, which stimulates mitochondrial fusion and elongation. In this process, it positively affects the activation of the RLR signalling pathway. Moreover, MFN1 knockdown causes inhibition of IFNβ and NF-κB promoter activation caused by mitochondrial fragmentation (39). In addition, MAVS redistribution has been shown to be induced by various viral infections and 5′-ppp-RNA transfection and is dependent on functional MFN1 (40).

#### 3.1.2 STING (MITA)

STING (MITA, mediator of IRF-3 activator) is a protein that binds to MAVS and is crucial for IFN production and NF-κB activation after viral infection. The endoplasmic reticulum (ER) was found to be more associated with elongated mitochondria during infection compared to control cells. This enabled the hypothesis that elongation of mitochondria after RLR activation promotes MAVS binding to the ER factor required for signaling. It is suggested that due to the formation of MAVS-STING and MAVS-MFN1 complexes, at the site of ER binding to the mitochondrion, MAVS is activated and transmits the signal further for type I IFN and cytokine production (39).

#### 3.1.3 Ankrd17

Proteins with ankyrin repeats are involved in various physiological processes, such as cell cycle control, transcriptional regulation and inflammatory responses (41). Ankrd17 belongs to a family of such proteins and consists of 25 ankyrin repeats at its N-terminus, which are divided into two clusters by a linker region. For some time, it was only known to be involved in cell cycle regulation (54). Over time, however, it was discovered that ankrd17 interacts strongly with MAVS by enhancing its interaction with RIG-I and MDA5. In addition, ankrd17 was found to coordinate with VISA to increase the expression of ISRE, NF-κB and IFN-β reporters without affecting cell viability. Activation of ISRE (IFN-stimulated response elements), NF-κB and IFN-β promoters is mediated by RIG-I. Moreover, it was also observed that ankrd17 enhanced MDA5-mediated activation and promoted MITA-mediated activation of the ISRE reporter (41).

#### 3.1.4 Ubiquitination

As previously mentioned, ubiquitination plays an important role in the activation of the RLR receptor-dependent signalling pathway. It provides specificity and regulates the intensity of innate immunity. Ubiquitination is a post-translational protein modification (PTM) that involves the covalent attachment of the some protein ubiquitin to target proteins (55). Ubiquitination is catalyzed by ubiquitin activating enzyme (E1), ubiquitin-conjugatin enzyme (E2) and ubiquitin protein ligase (E3). Ubiquitin can undergo ubiquitination itself on its seven lysine residues (K6, K11, K27, K29, K33, K48 and K63), building lysine-linked polyubiquitin chains (48). Unfolded CARD RIG-I undergoes tetramerization upon K63-bound polyquitination or upon attachment of an unanchored polyquitin chain (56). These modifications contribute to the accumulation of RIG-I, which promotes the interaction of the CARD RIG-I domain with the CARD MAVS domain and induces its oligomerization and fibrosis. Oligomerization and polyubiquitination stabilize activated CARD RIG-I due to the high amount of hydrophobic residues in this domain (48).

The first known and described site at which RIG-I undergoes ubiquitination is K172, and it is dependent on the E3 activity of the three-element motif 25 (TRIM25) protein (31). In addition, three CARD residues K154, K164 and K172 are ubiquitinated by gene (RING) finger protein-135 (RNF135) (42). RNF135 enables activation of CARD RIG-I by TRIM25 after ubiquitination of RD residues K849 and K851. Knockdown of RNF135 inhibits the interaction between RIG-I:TRIM25 and eliminates TBK1 recruitment (57).

#### 3.1.5 RAVER1

Another protein that participates in the regulation of RLR receptors and more specifically MDA5 is RAVER1 (ribonucleic PTB-binding 1). RAVER1 is specifically bound to MDA5, and its dormancy inhibits type I IFN induction *via* this receptor, but not *via* RIG-I. Mechanistically, RAVER1 promotes MDA5 binding to poly(I:C), and its knockdown inhibits activation of ISRE, NF-κB and the IFN-β promoter (43).

### 3.2 Negative regulators of RLR

#### 3.2.1 DUBs

As stated before, ubiquitination is pivotal for activating the antiviral immune response. However, it should be emphasized that ubiquitin (Ub) chains can also be remodeled by deubiquitinating enzymes (DUBs). This regulates the function and abundance of proteins involved in the regulation of innate immunity (48). Viruses use post-translational modifications to degrade various viral and cellular proteins to overcome host defense mechanisms at various stages of infection. They use DUBs to reverse the biological effects of ubiquitinated proteins by removing Ub from target proteins during viral infection of the host (58). DUBs such as USP3, USP15, USP21 and cylindromatosis (CYLD) are involved in type I IFN inhibition by directly targeting the RIG-I receptor causing K63 deubiquitination (44–47). In addition, cellular DUBs may be involved in altering the infectivity, replication, and pathogenicity of the virus. USP14 increases the replicability of a panel of viruses such as encephalomyocarditis virus, Sindbis virus and La Crosse virus and Epstein-Barr virus (EBV) uses USP7 to initiate disruption of PML nuclear bodies (59, 60). However, not all DUBs negatively affect the activation of the RLR-dependent signaling pathway. Some may have beneficial effects, for example, by enhancing NF-κB activity in the TLR pathway or stabilizing RIG-I by removing the poly-Ub K-48 chain (61, 62).

#### 3.2.2 RNF125 and LUBAC

RLR signaling can also be reduced by modifying Ub. Involved in this is RNF125 (ring-finger protein 125, called also TRAC-1 [T cell RING protein identified in activation screen]), which binds K48-linked polyubiquitin chains to CARD-activated RIG-I and MDA5, leading to degradation of both receptors and impairment of IFN-I signaling (48). RNF125 also ubiquitinates and degrades activated MAVS suggesting that it is capable of destabilizing the protein containing activated CARD (49). Because the CARD domain is often found in proteins involved in immune signaling pathways, RNF125 is considered an antagonist of immune signaling (63).

Another factor that negatively affects RIG-I receptor activation is the linear ubiquitin complex (LUBAC), which contains the IRP2 ubiquitin ligase 1L (HOIL-1L) and the HOIP protein, which interacts with HOIL-1L. These two structures regulate type I IFN expression through two mechanisms that act independently of each other (64). HOIL-1L competes with TRIM25 for RIG-I CARD binding, abolishing the RIG-I:MAVS interaction. In turn, HOIP, promotes M1- and K48-linked polyubiquitination of TRIM25 and induces its proteasomal degradation. Thus, TRIM25-mediated activation of RIG-I is reduced (48).

#### 3.2.3 NLRX1

NLRX1 belongs to the NLR family of receptors, which are also involved in the antiviral immune response. NLRX1 is localized in the outer mitochondrial membrane. It has been found to compete with activated RIG-I and MDA5 for binding of the CARD domain of the MAVS protein resulting in inhibition of type I IFN secretion (48). The C-terminal domain of the LRR of the NLRX1 receptor interacts with the CARD domain of MAVS blocking its activation and signal transduction involving the RIG-I or MDA5 receptors (50).

#### 3.2.4 A20 and TAX1BP1

A20 (also called TNFAIP3) is a ubiquitin-modifying enzyme that down-regulates antiviral signaling pathways that lead to IRF3 activation (51). The mechanisms by which A20 inhibits the antiviral response are poorly described; however, a protein that interacts with A20, TAX1BP1 (Tax1 binding protein 1, also known as T6BP or TXBP151), has been identified as a negative regulator of antiviral signaling pathways (52). A20 and TAX1BP1 work together to interrupt antiviral signaling. This is done by antagonizing Lys^63^-linked polyubiquitination of TBK1 and IKKi. A20 is unable to interact with TBK1 or IKKi in the absence of TAX1BP1, so TAX1BP1 is thought to act as an adaptor molecule for A20. In addition, A20 has been shown to block signaling to IRF3. Moreover, TAX1BP1 expression was shown to strongly block IFNβ activation mediated by virus infection, and TAX1BP1 overexpression blocked NF-κB activation (52, 65, 66).

#### 3.2.5 Atg5-Atg12

The role of autophagy in viral infection and activation of the RLR-dependent antiviral response is not fully elucidated (53). Autophagy is an essential process for physiological homeostasis, and is also involved in the elimination of some intracellular bacteria, such as invasive group A *Streptococcus*, *Mycobacterium tuberculosis* and *Shigella flexneri* (67–69). Studies have shown that members of the Atg family associated with autophagy (LC3, Atg5 and Atg12) co-localize with double-membrane cytoplasmic vesicles, where the viral RNA replication complex accumulates and initiates replication of the viral genome. The Atg5-Atg12 conjugate has been shown to interact directly with MAVS and RIG-I through CARD domains. This results in inhibition of type I IFN production and allows the virus to replicate in cells (53). LRRC25 (leucine-rich repeat containing protein 25) was also shown to bind to ISG15-modified RIG-I facilitating the interaction between RIG-I and the autophagic cargo receptor p62. This causes selective autophagy of RIG-I, resulting in reduced type I IFN production (70). Some viruses also cooperate with autophagy and weaken the immune response. It has been confirmed that human parainfluenza virus type 3 (HPIV3) can induce mitophagy, resulting in the degradation of MAVS and a reduction in the IFN response, in which RLRs are involved (71). Another study showed that Beclin-1 also interacts with MAVS CARD by blocking RIG-I signaling (72). Moreover, both increased levels of mitochondrial mass were observed and a higher percentage of cells that accumulated damaged mitochondria were detected in Atg5-deficient cells. This results in higher MAVS expression and thus increased signaling through the RLR. In addition, mitochondria are major producers of reactive oxygen species (ROS). The lack of autophagy contributes to the lack of removal of damaged mitochondria, which represent an additional source of ROS in the cell. It has been confirmed that an increase in ROS levels in the absence of autophagy results in RLR stimulation and increased IFN production. These results allow us to conclude that the absence of autophagy leads to an enhancement of RLR signaling in two ways. First, mitochondria accumulate in the cell, leading to the accumulation of MAVS, which is a key signaling protein for RLR. Second, damaged mitochondria that do not degrade are a source of ROS, which enhance RLR signaling in Atg5 knockout cells (73).

## 4 Mechanism of action of RLR in viral hemorrhagic fever

**Figure 3:**
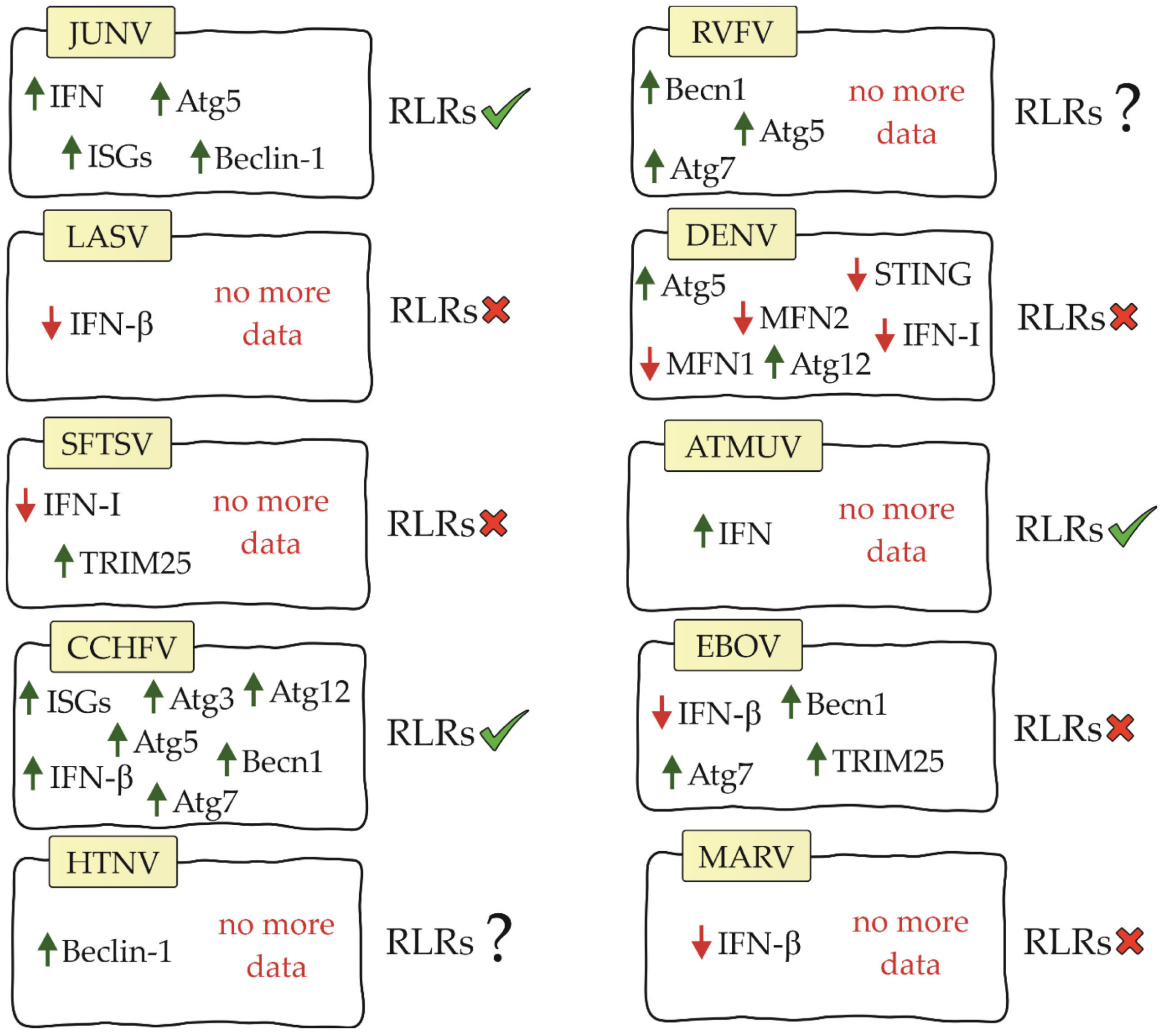
Diagram showing relationship between RIG-I-like receptors, their factors and viral hemorrhagic fevers (JUNV, Junín virus; LASV, Lassa virus; SFTSV, severe fever with thrombocytopenia syndrome virus; CCHFV, Crimean-Congo hemorrhagic fever virus; HTNV, Hantaan virus; RVFV, Rift Valley Fever Virus; DENV, dengue virus; ATMUV, avian Tembus virus; EBOV, Ebola virus; MARV, Marburg virus; RLRs, RIG-I-like receptors).

Viral hemorrhagic fever (VHF) is a group of acute zoonotic diseases with high mortality rates that infect both humans and animals. These diseases are endemic in some parts of the world and can cause serious outbreaks. Due to the poor prognosis and lack of specific vaccines or drugs, VHF remains a serious health problem worldwide. Understanding the pathogenesis of VHF disease can provide effective means to treat and monitor disease outcomes (74). The US Institute for Infectious Disease Medical Research lists four families of RNA viruses as the main etiological agents of VHF: *Arenaviridae*, *Bunyaviridae*, *Filoviridae* and *Flaviviridae* (75). Information on RIG-I-like receptors and the factors affecting them is residual. The following is the current state of knowledge in this field (**Figure 3**).

### 4.1 Arenaviridae

Arenaviruses belong to the enveloped viruses whose genetic material is the (–)ssRNA molecule. The arenavirus genome is two-segmented and consists of one large segment (L) of about 7.2 kb and one small segment (S) of about 3.4 kb. The S segment is responsible for encoding the viral glycoprotein (GP) precursor, which is post-translationally cut into a stable signal peptide (SSP) and mature GP1 and GP2. GP1 and GP2 form spikes located on the surface of the virus, which are responsible for binding to host receptors and mediating entry into cells. The S segment also encodes a nucleoprotein (NP), which is the major structural component of the nucleocapsid and is the most abundant protein during infection. The L segment encodes the RNA-dependent RNA polymerase L protein and a small, zinc finger protein (Z), which is responsible for virus particle formation (76, 77). All human pathogens in the family *Arenaviridae* belong to the genus *Mammarenavirus*, which we can further divide into two groups based on geography and phylogeny: Old World (OW) arenaviruses and New World (NW) arenaviruses (78, 79).

Both ends of the arenavirus genome form structures that contain 5’ppp acting as PAMPs (77). Studies on the Junín virus (JUNV), which belongs to the NW arenaviruses, have shown that dsRNA-derived signalling is in the same location as RIG-I and MDA5, suggesting that RLRs are involved in the recognition of arenavirus infection (80). Furthermore, IFN and interferon-stimulated genes (ISGs) were shown to be up-regulated during infection with this virus (81, 82). The use of siRNA to knockdown RIG-I resulted in a decrease in IFN-β and ISG. These results confirm that IFN pathway activation is RIG-I receptor-dependent in JUNV infection (83). Autophagy also occurs in JUNV infection. Studies have shown that the autophagy proteins Atg5 and Beclin-1 are essential for virus replication (84). These results contradict previously published information about the negative effects of these proteins on RLR receptor activation. This is an interesting aspect that certainly requires further analysis to better understand the impact of autophagy on the initiation of innate immunity.

However, the Lassa virus (LASV), which is classified as an OW arenavirus, has been shown to develop the ability to evade the RIG-I response (85). IFN-β is not increased during LASV infection, suggesting that this virus either avoids or inhibits the signalling pathway leading to IFN production (82). Furthermore, studies on myeloid dendritic cells showed that LASV does not increase MDA5 gene expression (86).

### 4.2 Bunyaviridae

Bunyaviruses are a family of spherical, enveloped viruses. They contain three segments of antisense (and sometimes ambisense) single-stranded RNA linked to a nucleoprotein (76). The two outer glycoproteins form projections on the surface of the virus. A virus-encoded transcriptase is present in the virion. Bunyaviruses replicate in the cytoplasm. Their RNA genome is transcribed into mRNA. The host RNA sequence in some representative viruses initiates the synthesis of viral mRNA. Bunyaviruses mature by budding into vesicles in or near the Golgi apparatus. Bunyaviruses are responsible for many diseases that run feverishly in humans and other vertebrates (87).

Studies on severe fever with thrombocytopenia syndrome virus (SFTSV) have shown that its non-structural protein can antagonize the RIG-I and MDA5 signalling cascade by targeting the downstream kinases TBK1/IKKϵ and inhibiting type I IFN production. These results suggest that RLRs are involved in the immune response during SFTSV infection (88–91). Other studies have shown that SFTSV infection results in increased mRNA and protein expression of RIG-I and MDA5. However, it does not increase the expression of MAVS. Furthermore, knockdown of MAVS results in blockade of IFN-β and NF-κB promoter activation stimulated by SFTSV (92). Its non-structural protein can also bind and sequester TRIM25, blocking its functions (93).

Another virus in the *Bunyaviridae* family, Crimean-Congo hemorrhagic fever virus (CCHFV), has a genome with a 5’ monophosphate (5’p) end (94). Because RIG-I recognizes only the 5′ppp and 5′pp ends, it is considered not to be involved in initiating the immune response upon CCHFV infection. However, our study supports the role for RIG-I in the antiviral response of CCHFV. RIG-I has been shown to be involved in increasing ISG expression and IFN-β production in CCHFV infection. Furthermore, knockdown of RIG-I resulted in an average increase in viral titer of 3.95-fold and 3.75-fold, respectively. This demonstrates that RIG-I also has an effect on virus replication (95). CCHFV infection is also characterized by a slight increase in autophagy proteins (Atg5, Atg7, Atg3, Atg12 and Becn1) (96). However, they have been shown not to affect RLR receptor activation. As in the case of JUNV infection, studies on the mechanisms affecting RLR receptors in these virus infections may be key to understanding the relationship between these mechanisms.

To date, there are no studies available on RIG-I-like receptors in infection with other hemorrhagic fever viruses of the *Bunyaviridae* family. However, there are studies on Hantaan virus (HTNV) that have shown that this virus manipulates the autophagic process to its advantage. During HTNV infection, mitophagy occurs (97). As we described earlier, this process affects RLR-dependent inhibition of IFN production. In addition, Beclin-1 knockdown inhibits HTNV replication (76, 97), so we speculate that this phenomenon may also be related to the RIG-I-like receptor signaling pathway and interferon production. In Rift Valley Fever Virus (RVFV) infection, silencing of autophagy proteins (Atg5, Atg7 and Becn1) has been shown to negatively affect the host antiviral response and increase viral replication (98). We suppose that in this case the situation may be similar to JUNV and CCHFV infection and the autophagy process does not negatively affect RLR signaling. However, confirmation of this theory requires studies.

### 4.3 Flaviviridae

*Flaviviridae* are a family of viruses whose genetic material is the (+)ssRNA molecule. They encode at least three structural proteins such as C (capsid), M/prM (vestibule) and E (envelope) and seven non-structural proteins (NS1, NS2A, NS2B, NS3, NS4A, NS4B and NS5) (76, 99). Flavivirus contains more than 70 viruses, including dengue virus (DENV), West Nile virus (WNV), Zika virus (ZIKV), Japanese encephalitis virus (JEV), and avian Tembus virus (ATMUV) (100, 101).

In DENV studies, it was demonstrated that 5′-pppRNA effectively initiated an antiviral response that was dependent on the RIG-I/MAVS/TBK1/IRF3 pathway and contributed to the reduction of infection (102). Te nonstructural proteins (NS2A and NS4B) of DENV serotype 4 (DENV4) were shown to inhibit IFN-β secretion that is induced by RIG-I-, MDA5-, MAVS-, and TBK1/IKKϵ. However, it is noteworthy that blocking the pathway does not occur by affecting the RLR receptors themselves, but by inhibiting the phosphorylation of TBK1 and IRF3, that is, at the level of activation of the TBK1 complex (103). In the case of DENV infection, there is again a pattern where RLR receptor activation is noted in parallel with autophagy supporting viral replication. During infection, increased expression of Atg5 and Atg12, potential inhibitors of RLR receptors, was demonstrated (104). In addition, the DENV NS2B3 protease cleaves MFN1 and MFN2 preventing proper mitochondrial function (105). The same protease can inhibit type I IFN production by cleaving human STING (74).

For other viruses in the Flaviviridae family, ATMUV has been shown to increase MDA5 expression, and silencing of MDA5 contributes to a significant decrease in IFN production. Based on these data, MDA5 has a key role in activating the immune response in ATMUV infection; however, the role of RIG-I has not been elucidated (106). RIG-I also recognizes JEV and secretes inflammatory factors (IL-6, IL-12p70, MCP-1, IP-10, and TNF- α) that help fight the virus (107).

### 4.4 Filoviridae

Members of the *Filoviridae* family are (–) ssRNA viruses, which include three genera: *Ebolavirus*, *Marburgvirus*, and *Cuevavirus*. They encode at least four proteins that counter host antiviral defense strategies such as glycoprotein (GP) and the viral proteins (VP) VP24, VP35, and VP40 (76, 108).

VP35 filovirus proteins are multifunctional. One of their functions is to counteract the induction of antiviral response. Ebola virus (EBOV) and Marburg virus (MARV) VP35 proteins bind viral dsRNAs to prevent their recognition by RIG-I and MDA5 (109, 110). A number of conserved basic residues facilitate the binding of EBOV VP35 to the phosphodiester backbone of dsRNA thereby inhibiting IFN secretion (110, 111). Some viral proteins such as EBOV and MARV VP35 inhibit RIG-I activation by targeting PACT, which induces activation of RIG-I-dependent IFNβ promoter activity. Expression of the C-terminal domain of EBOV VP35 inhibits RIG-I ATPase activity as well as IFNβ promoter activity (112, 113). EBOV VP35 also inhibits signalling mediated by IRF3 and IRF7. It partially binds to and inhibits the function of upstream kinases TBK1 and IKKϵ. MARV VP35 also inhibits IRF3 phosphorylation and IRF3 reporter gene activity, even in the presence of TBK1 and IKKϵ overexpression (109). Inhibition of RIG-I and MDA5 receptor activation in EBOV infection may be caused by autophagy. Studies have shown that autophagy proteins are necessary for the virus to enter the host *via* macropinocytosis. In addition, deletion of Becn1 and ATG7 was confirmed to block virus entry (76, 114). However, TRIM25 was found to inhibit EBOV transcription and replication through inhibition of virus-like frequency (trVLP) propagation. TRIM25 interacts with the viral helical ribonucleoprotein complex, causing its auto-ubiquitination and ubiquitination of the viral nucleoprotein. As can be seen, this process is independent of RIG-I, so in this case TRIM25 does not act on RLR receptors as a positive factor (115).

## 5 *Lagovirus europaeus*

VHF is characterized by a difficult diagnostic process and a lack of effective treatments, so they pose a public health challenge, especially in subtropical regions. High virulence and rare outbreaks occurring in geographically remote areas with poor diagnostic facilities are the main factors making VHF analysis in humans ineffective (116). Therefore, animal models are a key element for advancing knowledge of VHF and establishing new therapeutic and diagnostic strategies (76). A good VHF model should meet the following criteria: uncontrolled viral expansion into multiple organs, viral suppression of the type I interferon response, triggering the secretion of large amounts of pro-inflammatory cytokines, induced primary infection of monocytes/macrophages and dendritic cells, and liver damage caused by infection of Kupffer cells and hepatocytes (117). We believe that *Lagovirus europaeus* infection shares many characteristics with VHF and probably it makes this virus a very good animal model for testing to other viral hemorrhagic fevers.

*Lagovirus europaeus*/GI.1 is highly pathogenic virus that causes rabbit hemorrhagic disease (RHD) in both, wild and domestic European rabbits (*Oryctolagus cuniculus*). It belongs to the *Caliciviridae* family, and was first detected in 1984 in China (118). *L. europaeus*/GI.2 first appeared in 2010 in France. It can infect both European rabbits and other hare species (*Lepus*). Among other things, innate immunity plays an important role in *Lagovirus europaeus* infection (119). One of the characteristic features of the virus is that non-young rabbits are resistant to infection until 4 weeks of age (GI.2) and 9 weeks of age (GI.1), respectively. Only 3% of adults survive *Lagovirus europaeus* infection. Such a high mortality rate and the rapid spread of RHD worldwide prompted work on a vaccine. However, to date, there is no system that allows *in vitro* culture of *Lagovirus europaeus* strains. For this reason, vaccines are based on inactivated virus isolated from the livers of infected rabbits. Current vaccines contain one or two antigens from both strains of the virus, as it has been proven that vaccination for *Lagovirus europaeus* confers limited cross-immunity to the other strain and vice versa (119). *L. europaeus*/GI.1 and GI.2 infection can occur in three clinical forms:subacute, acute, and chronic. RHD is characterized by acute fulminant hepatitis, splenomegaly, hemorrhage and congestion of several organs such as trachea, lungs, heart and kidneys, mainly associated with massive disseminated intravascular coagulation (120). Postmortem imaging shows acute hepatitis and an enlarged spleen (121). In addition, the virus also attacks macrophages and monocytes of the lungs, lymph nodes and monocytes located inside the liver vessels (122). Kupffer cells have been proven to be involved in viral replication and are presumably involved in the spread of virus particles in the body (123). Pathology after *L. europaeus* infection is characterized by two main processes (119). The first is related to impaired physiology with severe organ destruction (liver, spleen, kidney) and rapid viral replication (124). Infected rabbits show the presence of the virus in periventricular hepatocytes and macrophages of the liver, lungs and spleen during immunohistology and *in situ* hybridization (ISH). As the disease progresses, increased apoptosis of hepatocytes and liver endothelial cells is noted (121, 125). Multiorgan dysfunction is identified by respiratory acidosis, hypoglycemia and increased creatinine kinase activity (119). The second process involves an impaired immune response with induction of systemic lymphocyte apoptosis. Apoptosis of B and T lymphocytes in the liver and peripheral blood combined with neutrophilic infiltration results in decreased numbers of regulatory T lymphocytes and severe leukopenia before death (126–129). Increased expression of pro-inflammatory cytokines in the liver, spleen and serum of rabbits has also been demonstrated during infection (129).

Another important aspect is that RLR receptors have not yet been studied in *Lagovirus europaeus* infection, so this could be a good start for research using this virus. However, there are few data on autophagy in this infection. The increase in autophagy proteins such as Beclin-1, Atg12, Atg5 and LC3 suggests that there should be inhibition of the RIG-I-like receptor-dependent signaling pathway (130). However, cases such as DENV, RVFV, CCHFV and JUNV infection show that these mechanisms can occur in parallel and not affect each other. Therefore, conducting studies on *L. europaeus* infection may contribute to a deeper understanding of the relationship between these mechanisms and the pathogenesis of these infections. A routine diagnostic tool in *L. europaeus* infection is real-time PCR. This method has been shown to be 100% sensitive and to detect 10 copies of the virus per well. The test uses a liver fragment from an infected rabbit, from which a homogenate is then made (131).

## 6 Conclusion

In this review, we described the role played by RLR receptors in activating the antiviral response using viral hemorrhagic fevers as an example. We showed that the course of the disease depends on whether RLR receptors are activated, and IFN production occurs. However, the activation of RIG-I and MDA5 is influenced by many factors, including autophagy, which appears to be crucial in VHF, as viruses can use it as a strategy to evade the immune response (76). Just as it seems clear that RLR receptors play a key role in combating VHF, the mechanisms of receptor action and the factors affecting their activation have not been fully described. There is little data on RLR receptors in the VHF, and we know even less about how positive and negative factors can affect them. Further studies are needed to explore and describe these mechanisms. Given that there is an ongoing need for more effective treatment strategies for viral hemorrhagic fevers, the study of RLRs and the factors that regulate their function seems promising. However, due to the difficulties associated with studying VHFs in humans, we propose Lagovirus europaeus as an animal model, whose characteristics indicate that it meets the conditions we outlined earlier.

## Author contributions

All authors listed have made a substantial, direct, and intellectual contribution to the work and approved it for publication.

## Conflict of interest

The authors declare that the research was conducted in the absence of any commercial or financial relationships that could be construed as a potential conflict of interest.

## Publisher’s note

All claims expressed in this article are solely those of the authors and do not necessarily represent those of their affiliated organizations, or those of the publisher, the editors and the reviewers. Any product that may be evaluated in this article, or claim that may be made by its manufacturer, is not guaranteed or endorsed by the publisher.
